# Indications of flat bands driving the *δ* to *α* volume collapse of plutonium

**DOI:** 10.1073/pnas.2308729121

**Published:** 2024-02-14

**Authors:** Neil Harrison, Greta L. Chappell, Paul H. Tobash

**Affiliations:** ^a^MPA-MAGLAB, Los Alamos National Laboratory, Los Alamos, NM 87545; ^b^MST-16, Los Alamos National Laboratory, Los Alamos, NM 87545

**Keywords:** plutonium, entropy, structural transition, volume collapse, flatbands

## Abstract

On cooling from the melt, plutonium (Pu) undergoes a series of structural transformations accompanied by a ≈ 28% reduction in volume from its *δ* phase to its *α* phase at low temperatures. While Pu’s partially filled 5*f*-electron shells are known to be involved, their precise role in the transformations has remained unclear. By using calorimetry measurements on *α*-Pu and gallium-stabilized *δ*-Pu combined with resonant ultrasound and X-ray scattering data to account for the anomalously large softening of the lattice with temperature, we show here that the difference in electronic entropy between the *α* and *δ* phases dominates over the difference in phonon entropy. Rather than finding an electronic specific heat characteristic of broad *f*-electron bands in *α*-Pu, as might be expected to occur within a Kondo collapsed phase in analogy with cerium, we find it to be indicative of flatter subbands. An important role played by Pu’s 5*f* electrons in the formation of its larger unit cell *α* phase comprising inequivalent lattice sites and varying bond lengths is therefore suggested.

## Significance

The structural transformations of plutonium (Pu) upon cooling from the melt, specifically from the *δ* to the *α* phase, have long puzzled researchers. In this study, we employed calorimetry, resonant ultrasound, and X-ray scattering to elucidate the underlying mechanisms. Our findings reveal that the dominant factor driving these transformations is the difference in electronic entropy between the *α* and *δ* phases, rather than phonon entropy. Surprisingly, unlike expectations based on analogous systems, *α*-Pu exhibits a specific heat characteristic indicative of flatter subbands instead of broad *f*-electron bands. These results suggest a crucial role for Pu’s 5*f* electrons in the formation of the *α* phase, with its distinct unit cell and varying bond lengths. This understanding enhances our knowledge of Pu’s complex behavior and contributes to the broader understanding of *f*-electron systems.

**Fig. 1.**
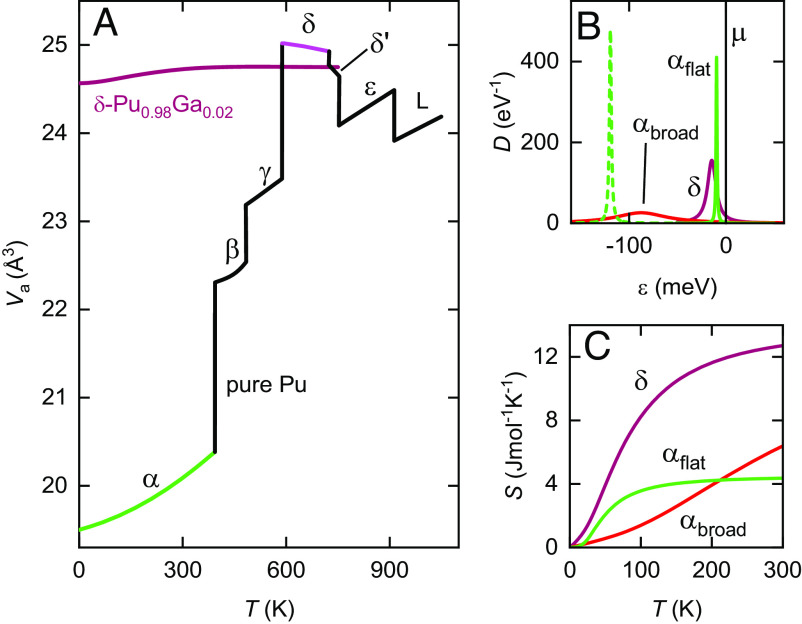
Schematic of volume, electronic DOS and electronic entropy. (*A*) Atomic volume $V_{a}$ of Pu from various thermal expansion and phase transition measurements (black curve) (5). Greek letters refer to the allotropes of Pu while “L” refers to the liquid. The *α* and *δ* phases are highlighted in different colors. *δ*-Pu_0.98_Ga_0.02_ is also shown for comparison (determined instead using neutron scattering measurements) (32). (*B*) $\alpha_{\mathrm{flat}}$ and *δ* shows the peaks in the electronic DOS required to account for features observed in the specific heat resembling Schotte–Schotte anomalies. $\alpha_{\mathrm{broad}}$ shows schematically the broader peak that would be expected in a Kondo collapse scenario. (*C*) The corresponding entropy *S* curves.

All elements are known to undergo structural transformations under extreme environments of pressure and temperature, leading to phase diagrams consisting of multiple crystalline phases (1). In the actinide series, unusually complex multiphase phase diagrams occur even in the absence of pressure (2, 3). Among these, elemental plutonium (Pu) is exceptional in that six different solid state allotropes are accessible from ambient conditions by increasing the temperature to ∼750 K (4, 5) (Fig. 1*A*). However, the fundamental mechanism responsible for driving its multiple structural transformations has remained elusive. In the vast majority of materials, it is the phonon entropy that is the primary thermodynamic driver of structural transformations with increasing temperature (6, 7). Softer phases contain more vibrational entropy than stiffer phases, causing them to be favored at higher temperatures. However, the presence of partially filled 5*f*-electron shells in Pu implies that these can also contribute significantly to the entropy by the formation of narrow electronic bands close to the chemical potential (8).

One widely held view is that the reduction in volume from *δ*-Pu to *α*-Pu is primarily driven by a mechanism (8–10) analogous to the Kondo volume collapse or Mott localization mechanisms proposed for 4f electrons in cerium (Ce) (11, 12). In the former model, the collapse is associated with a large increase in the Kondo coupling, while in the latter, it is linked to a transformation of the *f* electrons from localized to itinerant states. In this context, the inverse relationship between volume and electronic bandwidth resembles that between volume and phonon bandwidth, resulting in broader electronic bands in smaller volume phases. Consequently, the reduced entropy of broader bands in smaller volume *α*-Pu has been suggested as a possible explanation for why this phase, rather than *δ*-Pu, is more stable at low temperatures, similar to the situation with the smaller volume *α* phase of Ce (13, 14).

In the case of Ce, where the volume collapse is isostructural, both the Kondo volume collapse and Mott localization mechanisms offer viable explanations for the physics of that system. However, when considering systems like Pu, where the volume collapse is accompanied by structural transformations, the applicability of these models becomes highly uncertain.

Given the martensitic nature of the transformations in Pu (3) and the formation of long and short bonds (15) in *α*-Pu, an alternative hypothesis is that the reductions in crystalline symmetry can be regarded phenomenologically as Peierls-type instabilities (16, 17). Whereas *δ*-Pu has a face-centered cubic structure with a single lattice site, *α*-Pu has a low symmetry monoclinic crystalline structure comprising eight distinct lattice sites. One general consequence of a larger more complex unit cell comprising inequivalent sites (18–20) is that it opens gaps in the electronic density of states, producing subbands that are much flatter (21–23) and sharper peaks in the electronic density of states. Recent electronic structure calculations have suggested that flat 5f-electron bands can indeed occur within the larger more complex unit cell phases Pu (24).

In this paper, we show that we can distinguish between scenarios in which the electronic bands in *α*-Pu become broader or flatter relative to those in *δ*-Pu by way of calorimetry measurements (25). We combine calorimetry measurements with a procedure in which we use X-ray scattering measurements of the phonon density of states (26, 27) and temperature-dependent resonant ultrasound (RUS) measurements (28) so as to accurately account for the anomalously large softening of the lattice with temperature in Pu (29). The leading electronic contributions to the specific heats of both *α*- and *δ*-Pu are found to resemble a Schotte–Schotte anomaly (30), which we attribute to a narrow 5f-electron peak close to the chemical potential (Fig. 1*B* and *Materials and Methods*). Consistent with the creation of flatter subbands in a larger unit cell in *α*-Pu, we find the anomaly in *α*-Pu to be narrower and of lower spectral weight (i.e., ≪1 electron per Pu site). We show that it is its lower spectral weight that reduces the electronic entropy of *α*-Pu relative to *δ*-Pu (illustrated in Fig. 1*C*), in contrast to a reduction in entropy caused by the formation of broader bands as in a Kondo collapse scenario (13, 14).

## Results

**Fig. 2.**
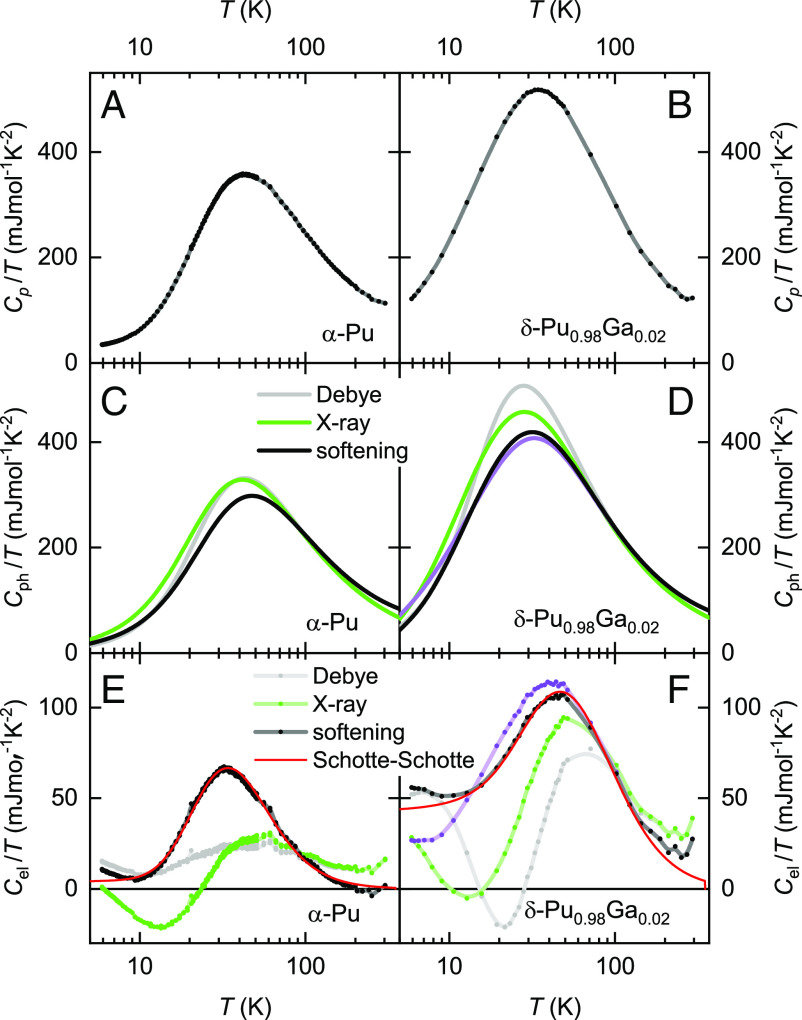
Calorimetry data. (*A*) Measured $C_{p}\left(T\right)/T$ of *α*-Pu. (*B*) Measured $C_{p}\left(T\right)/T$ of *δ*-Pu_0.98_Ga_0.02_. (*C*) Calculated $C_{\mathrm{ph}}\left(T\right)/T$ of *α*-Pu, using the Debye model (gray curve), the X-ray scattering phonon DOS neglecting phonon softening (green curve), and the X-ray scattering phonon DOS including phonon softening (black curve). (*D*) Similarly calculated $C_{\mathrm{ph}}\left(T\right)/T$ of *δ*-Pu_0.98_Ga_0.02_. Here, the black curve shows $C_{\mathrm{ph}}\left(T\right)/T$ obtained using ref. 26 (taking into consideration phonon softening), whereas the purple curve shows $C_{\mathrm{ph}}\left(T\right)/T$ obtained using ref. 27 (again, taking into consideration phonon softening). Note that a contribution from ∂β/∂T contributes only ∼ 10 mJmol^−1^K^−2^ to $C_{\mathrm{ph}}/T$ at 300 K in (*C*) and (*D*); its contribution to $\Delta S_{\mathrm{ph}}^{\delta -\alpha }$ is therefore small. (*E*) $C_{\mathrm{el}}\left(T\right)/T$ of *α*-Pu, obtained by subtraction, as described in the text. The red curve is a calculated Schotte–Schotte functional form with a spectral weight of η= 0.39, gap energy Δ= 9.7 meV and line width Γ= 0.61 meV. (*F*) Similarly subtracted $C_{\mathrm{el}}\left(T\right)/T$ of *δ*-Pu_0.98_Ga_0.02_. The red curve here is a calculated Schotte–Schotte functional form for η≈ 1.2, Δ= 14.6 meV and Γ= 5.3 meV, which has been matched to the black curve.

A crucial factor in us being able to distinguish between the broad band and flat subband scenarios depicted in Fig. 1 *B* and *C* is the higher signal-to-noise ratio of our specific heat $C_{p}$ (at constant pressure) data compared to those obtained in prior studies (31); the data are plotted as $C_{p}/T$ vs. temperature *T* in Fig. 2. Fig. 2*A* shows data obtained on double electro-refined *α*-Pu (*Materials and Methods*), while Fig. 2*B* shows our prior measurements of *δ*-Pu_0.98_Ga_0.02_ (2 atomic % Ga-stabilized *δ*-Pu) (25). Whereas the *δ* phase is stable only between ≈ 580 and 720 K in pure Pu (Fig. 1*A*), the substitution of Ga enables this phase to remain mostly stable (*Materials and Methods*) down to cryogenic temperatures (3) without its measured thermodynamic properties (volume, elastic moduli, thermal expansivity, specific heat, etc.; see e.g., Fig. 1*A*) departing significantly from those of pure *δ*-Pu (28, 32–34).

The extraction of the electronic contributions to the specific heat requires us first to make independent determinations of the phonon contributions in Fig. 2 *C* and *D*. We achieve this by using the expression (35)

**Fig. 3.**
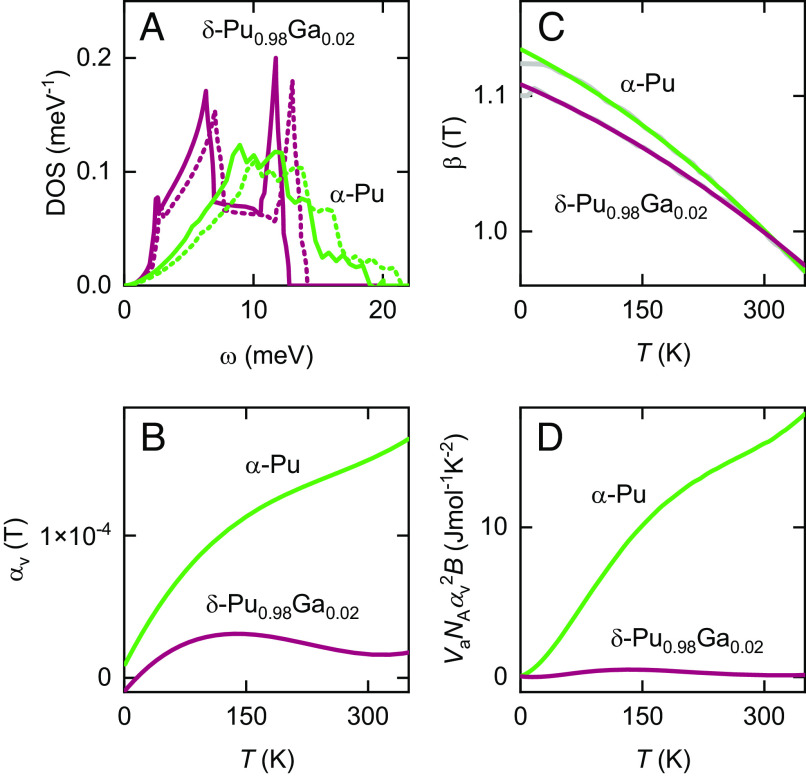
Other thermodynamic data used in the analysis. (*A*) Phonon DOS for *α*-Pu (27) and *δ*-Pu_0.98_Ga_0.02_ (26) obtained from X-ray scattering experiments. Solid lines are the measured DOS at room temperature, while the dotted lines correspond to the stiffer phonon DOS expected at zero temperature from changes in the sound velocity. (*B*) The volume thermal expansivity $\alpha_{v}\left(T\right)$ according to dilatometry and neutron scattering measurements (Fig. 1). (*C*) The phonon softening parameter β(T) obtained from RUS measurements (28). (*D*) $V_{a}N_{A}\alpha_{v}{\left(T\right)}^{2}B\left(T\right)$ vs. *T*, from which we obtain *γ*.

[1] $$\begin{array}{cc} \frac{C_{\mathrm{ph}}\left(T\right)}{T} & =3R\frac{\partial }{\partial T}[\int_{0}^{\infty }D_{\mathrm{ph}}\left(\omega ,T\right)\\ & \;\times \;[\left(n_{\mathrm{BE}}+1\right)\ln\left(n_{\mathrm{BE}}+1\right)-n_{\mathrm{BE}}\ln\left(n_{\mathrm{BE}}\right)]d\omega ], \end{array}$$

for the phonon-specific heat divided by temperature, which allows for a phonon density of states (DOS) $D_{\mathrm{ph}}\left(\omega ,T\right)$ that is temperature-dependent (Fig. 3*A*). Here, *ω* is the phonon frequency, $n_{\mathrm{BE}}={\left(e^{\frac{\omega }{k_{B}T}}+1\right)}^{-1}$ is the Bose–Einstein distribution function and $R=N_{A}k_{B}$ is the gas constant. Neutron scattering experiments on *δ*-Pu_0.95_Al_05_ (*δ*-Pu stabilized with five atomic % Al) have provided direct evidence for a continuous softening of the phonon DOS with temperature (29), which occurs in the *δ* phase in spite of its unusually small thermal expansivity (32) (Figs. 1 and 3B). While the lowest energy acoustic part of the phonon DOS is missing in the neutron scattering results (29), the main features in the phonon DOS can be seen to shift with temperature while it retains its overall shape, implying that its temperature-dependence

[2] $$\begin{array}{c} D_{\mathrm{ph}}\left(\omega ,T\right)\approx \beta^{-1}\left(T\right)D\left(\beta^{-1}\left(T\right)\omega \right) \end{array}$$

can be considered to depend primarily on a single parameter: $\beta \left(T\right)\approx c\left(T\right)/c_{\mathrm{RT}}$ (Fig. 3*C*) to leading order, where c(T) is the sound velocity, and $c_{\mathrm{RT}}$ is its value at room temperature. We can then determine $D_{\mathrm{ph}}\left(\omega ,T\right)$ by combining temperature-dependent RUS measurements of c(T) with X-ray scattering measurements of $D_{\mathrm{ph}}\left(\omega \right)$ made at fixed (room) temperature. Strengths of the above approach are threefold: i) the acoustic part of the phonon DOS is not missing in X-ray scattering experiments (26, 27) (Fig. 3A), ii) RUS and X-ray scattering measurements have been performed on both *α*-Pu (27, 28) and *δ*-Pu_0.98_Ga_0.02_, and iii) an approximate treatment of anharmonic phonon effects is implicitly included via a ∂β/∂T contribution resulting from the substitution of Eq. **2** into Eq. **1**. The measured forms of the phonon DOS can also be accurately modeled (36–39). We determine the temperature-dependent sound velocity from the measured adiabatic bulk modulus B(T) using $c\left(T\right)=\sqrt{B(T)/\rho (T)}$, where $\rho \left(T\right)=MN_{A}/V_{a}\left(T\right)$ is the density (32, 40), and *M* is molar mass.

**Fig. 4.**
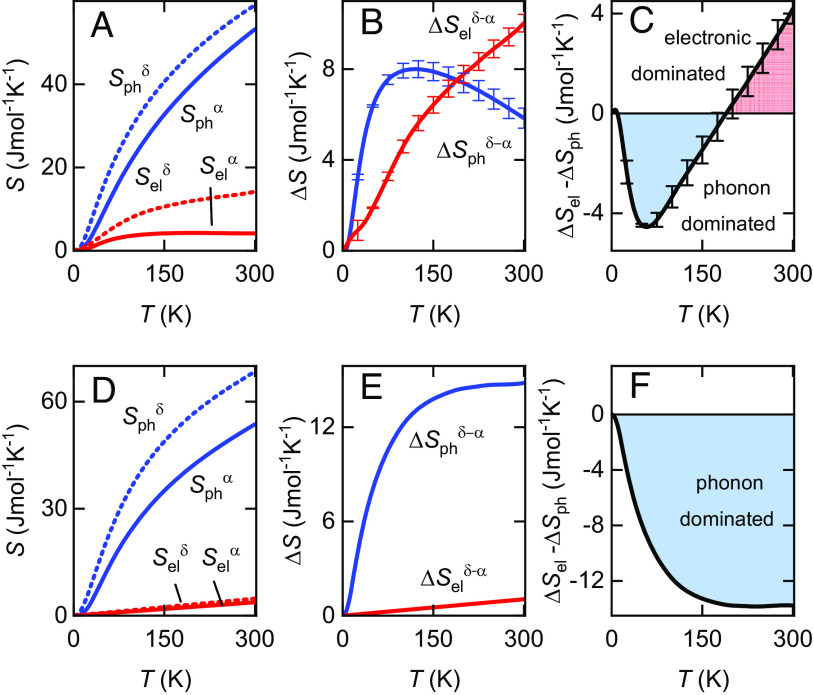
Entropy analysis. (*A*) Phonon and electronic contributions to the entropies of *α*-Pu and *δ*-Pu_0.98_Ga_0.02_, as indicated. (*B*) The difference in phonon and electronic entropy between *α*-Pu and *δ*-Pu_0.98_Ga_0.02_, as indicated. Since the overall softening (Fig. 3*B*) is similar for *α*-Pu and *δ*-Pu_0.98_Ga_0.2_, inaccuracies in the validity of Eq. **2** lead only to small systematic errors in $S_{\mathrm{ph}}\left(T\right)$ and $S_{\mathrm{el}}\left(T\right)$ (as indicated by error bars). (*C*) The difference between the electronic and phonon entropy differences. (*D*), (*E*), and (*F*) Same quantities assuming the electronic entropy contributions of Wallace (41) instead of those determined here.

Having obtained $C_{\mathrm{ph}}\left(T\right)$ (plotted as $C_{\mathrm{ph}}\left(T\right)/T$ in Fig. 2 *C* and *D*), the electronic contributions to the specific heat in Fig. 2 *E* and *F* are obtained using $C_{\mathrm{el}}\left(T\right)=C_{v}\left(T\right)-C_{\mathrm{ph}}\left(T\right)$, where $C_{p}\left(T\right)/C_{v}\left(T\right)=\gamma \left(T\right)$, which, in turn, is obtained using the thermodynamic identity $(\frac{\gamma (T)-1}{\gamma (T)})C_{p}=C_{p}\left(T\right)-C_{v}\left(T\right)=V_{a}N_{A}\alpha_{v}{\left(T\right)}^{2}B\left(T\right)T$ (Fig. 3*D*). Finally, we calculate the electronic and phonon contributions to the entropy in Fig. 4*A* using $S_{\mathrm{ph}}\left(T\right)=\int_{0}^{\infty }\left(\gamma \left(T\right)C_{\mathrm{ph}}\left(T\right)/T\right)dT$ and $S_{\mathrm{el}}\left(T\right)=\int_{0}^{\infty }\left(\gamma \left(T\right)C_{\mathrm{el}}\left(T\right)/T\right)dT$, under the assumption that the total is given by $S{\left(T\right)}_{\mathrm{tot}}=S_{\mathrm{ph}}\left(T\right)+S_{\mathrm{el}}\left(T\right)$.

Because the degree of phonon softening is very similar for *α*-Pu and *δ*-Pu_0.98_Ga_0.02_ in Fig. 3*C*, its effect on the relative sizes of the differences in electronic and phonon entropy between these phases is small (represented by error bars in Fig. 4 *B* and *C*). However, our ability to obtain physical estimates of $C_{\mathrm{el}}\left(T\right)/T$ in Fig. 2 is entirely conditional upon the effects of phonon softening being taken into consideration. Not accounting for these, either by using the fixed phonon DOS determined at room temperature from X-ray scattering experiments (green curves) or by resorting to the Debye model (gray curves), causes $C_{\mathrm{ph}}\left(T\right)/T$ to be overestimated at temperatures below ∼100 K in Fig. 2 *C* and *D*. This, in turn, leads to unphysical (negative) values of $C_{\mathrm{el}}\left(T\right)/T$ (represented by green and gray curves in Fig. 2 *E* and *F*). Only by performing a full integration using Eq. **1** that takes into account the phonon softening do we obtain physical (i.e., consistently positive) values of $C_{\mathrm{el}}\left(T\right)/T$ (represented by the black curves in Fig. 2 *E* and *F*).

The phonon dispersion characteristics of *δ*-Pu_0.98_Ga_0.02_ modeled in ref. 26 that we use in Fig. 2 remain a benchmark against which various theoretical approaches for calculating phonon dispersion have been evaluated (36, 37, 39). However, we find that the peak in $C_{\mathrm{el}}\left(T\right)/T$ remains consistent regardless of the choice of measured phonon density of states used for calculating $C_{\mathrm{ph}}/T$. Substituting the phonon density of states of *δ*-Pu_0.98_Ga_0.02_ from ref. 27 instead of ref. 26, we find a comparable $C_{\mathrm{ph}}/T$ (depicted by the purple curve in Fig. 2*D*). Utilizing this alternative $C_{\mathrm{ph}}/T$ to deduce $C_{\mathrm{el}}\left(T\right)/T$ (shown as the purple curve in Fig. 2*F*), we consistently observe a pronounced peak. The phonon density of states derived from ref. 27 intriguingly indicates softer phonons at lower energies, which leads to a reduced residual value of the Sommerfeld coefficient in Fig. 2*F*. However, since the integrated areas under the black and purple $C_{\mathrm{el}}\left(T\right)/T$ curves are nearly the same, the choice of phonon density of states has no significant impact on the entropy at high temperatures.

In both phases of Pu, we find that $C_{\mathrm{el}}\left(T\right)/T$ is close in functional form to a Schotte–Schotte anomaly (30). This result is physical because it is the functional form one obtains when the electronic DOS (closest to the chemical potential) consists of a Lorentzian situated above or below or symmetrically above and below (25) the chemical potential *μ* (as illustrated in Fig. 1*B*). It is the line-shape one obtains when a sharp feature in the electronic density of states is broadened by finite lifetime effects (*Materials and Methods*). Such a line-shape is consistent with a dominant peak that is predicted to lie close to *μ* in valence-fluctuating models of the electronic structure of *δ*-Pu (24, 42–44), although the width of the peak (2Γ≈ 11 meV) required to account for the experimental data in *δ*-Pu_0.98_Ga_0.02_ is smaller than the smallest width (≈ 70 meV) obtained thus far with theoretical modeling (15, 24, 42–44). A smaller experimental energy scale for *δ*-Pu in addition to that ∼100 meV associated with the invar effect (32) is supported by measurements of the Sommerfeld coefficient $\gamma_{\mathrm{el}}$ in the limit T→0 (25, 45, 46) and measurements of the magnetostriction coefficient as a function of temperature (47).

## Discussion

Were a Kondo collapse or Mott delocalization mechanism to prevail in Pu, we would expect the energy scales associated with the peaks exhibiting a Schotte–Schotte functional form in Fig. 2 to become significantly larger within the smaller volume *α* phase (8–10), as occurs in Ce (11–14). This corresponds to a broader peak in the electronic DOS (e.g., the red curve in Fig. 1*B*) and a reduced slope of the entropy with increasing temperature (red curve in Fig. 1*C*); for illustrative purposes, the red curves in Fig. 1 *B* and *C* correspond to a Kondo collapsed energy scale that is assumed to be six times larger than that of the *δ* phase, so as to be similar to the change in Kondo energy scale reported at the *γ* to *α* transition in Ce (12).

However, rather than finding broader bands, we observe the Schotte–Schotte functional form in $C_{\mathrm{el}}\left(T\right)/T$ for *α*-Pu (Fig. 2*E*) to be narrower than that in the *δ* phase (Fig. 2*F*). A narrower peak in $C_{\mathrm{el}}\left(T\right)/T$ indicates a narrower peak in the electronic density of states, which, in turn, suggests a flatter electronic band. While the narrower spectral feature close to the chemical potential in *α*-Pu compared to *δ*-Pu, as shown schematically in Fig. 1*B*, might initially suggest that *α*-Pu is comparatively less stable, its overall spectral weight is significantly reduced in comparison to *δ*-Pu. This reduction is evident in the entropy, involving an integration over these states as shown in Fig. 1*C*, or in the parameter *η* in the caption of Fig. 2. As a result, only a subset (≈30%) of the Pu atomic sites that are contributing to the *f*-electron states near the Fermi surface in *δ*-Pu continue contributing to a narrow spectral feature near the Fermi surface in *α*-Pu. Therefore, the relative internal energy of the *α* and *δ* phases will primarily depend on the fate of the remaining ≈70% of the *f*-electron states that no longer lie close to the chemical potential in *α*-Pu. A plausible explanation is that the ≈70% of remaining *f*-electron states reside in bonding and antibonding bands located further from the chemical potential in *α*-Pu (indicated schematically by a dashed green peak in Fig. 1*B*). If this holds true, these states must lie at least 100 meV from the chemical potential to avoid significant contributions to $C_{\mathrm{el}}/T$ at 300 K.

We can understand the above findings if we consider the changes in electronic structure that typically accompany a Peierls-like distortion of the lattice. While the overall atomic volume (per Pu site) of *α* is significantly reduced compared to *δ*-Pu (Fig. 1*A*), the extensively expanded unit cell comprising eight inequivalent atomic sites implies that the electronic structure of *α*-Pu encompasses a greater number of subbands. Not only must these subbands be flatter, as a result of the diminished size of the Brillouin zone, but an energy splitting is also anticipated between bonding and antibonding subbands due to the lattice changes accompanying the reduced symmetry of *α*-Pu relative to *δ*-Pu.

The relevance of a Peierls-like distortion has previously been discussed in connection with the lowest temperature, lowest symmetry *α* phases of uranium, neptunium, and Pu (16, 48). While strong electronic correlations are not necessary for the realization of a Peierls-like distortion in most instances, they increase the likelihood of a distortion by contributing to the narrowness of the electronic bands. Some qualitative support for such a scenario in *α*-Pu comes from very recent dynamic mean field theory (DMFT) calculations (24). In addition to finding sharper peaks close to the chemical potential than in *δ*-Pu, which are explicitly attributed to the formation of flat bands, extra peaks further above and below the chemical potential are shown to emerge in *α*-Pu that are absent in *δ*-Pu.

However, it is important to note that the positions (relative to the chemical potential) and the widths of peaks in the electronic density of states vary significantly among calculations of the electronic structure of Pu, pointing to a lack of consensus on the role of strong electronic correlations (15, 24, 42–44, 48). Furthermore, crystal electric field effects, which have yet to be fully incorporated into electronic structure models of Pu, could potentially also result in energy splittings of around 10 meV near the chemical potential (49, 50). A theoretical investigation that focuses on the microscopic processes governing the formation of bands near the chemical potential in *α*-Pu is therefore warranted.

In addition to the formation of bonding and antibonding bands in *α*-Pu offering a plausible method for reducing the internal energy of this phase compared to *δ*-Pu, it also significantly diminishes the electronic entropy as a function of temperature, as schematically shown in Fig. 1*C*. The higher electronic entropy of the *δ* phase favors the stability of this phase over the *α* phase at elevated temperatures. Electronic entropy is thus expected to play an equally crucial role in Pu as it does in Ce (14), albeit via a distinctly different mechanism.

Our finding of differences in the electronic structure between the *α* and *δ* phases near the chemical potential contrasts with the results obtained from photoemission measurements, which have shown surprisingly similar valence band spectra for these phases (9, 51). It has been argued that the resemblance observed in photoemission measurements may be caused by the surface of *α*-Pu having a tendency to undergo a reconstruction into a state resembling a *δ*-Pu structure (51). It is plausible, therefore, that our distinct findings in electronic properties between *α* and *δ* phases are due to calorimetry being largely unaffected by surface states.

The maxima in $C_{\mathrm{el}}/T$ in Fig. 2 *E* and *F*, along with the corresponding peaks in the electronic DOS as illustrated in Fig. 1*B*, bear a resemblance to those reported in other strongly correlated electron systems (52–54). This similarity suggests that both phases of Pu may be delicately poised near a secondary instability, such as the onset of magnetic order or superconductivity. Such ordered phases tend to emerge when the peak in the electronic DOS approaches the chemical potential. Theoretically, magnetism has been proposed as a mechanism for stabilizing the various phases of Pu (55), although experimental evidence for magnetic order remains elusive (56).

With regard to the Pu equation of state (57), our findings complement electronic structure calculations, which are considered to provide insight into the relative stability of the various phases of Pu at T=0 (55). Under the assumption that the *δ* and *α* phases preserve their electronic structure when doped with 2 atomic % Ga the latter usually referred to as $\alpha^{\prime }$-Pu when substituted with 2% Ga (3), our findings inform us of how the entropy in Pu should be accurately modeled as a function of temperature. Since $S_{\mathrm{el}}\left(T\right)\ll S_{\mathrm{ph}}\left(T\right)$ (Fig. 4*A*) in the vast majority of metals, it is usually assumed that a small constant $\gamma_{\mathrm{el}}$ for $C_{\mathrm{el}}\left(T\right)/T$ is an adequate approximation, corresponding to a simple linear form for the electronic entropy $S_{\mathrm{el}}\left(T\right)=\gamma_{\mathrm{el}}T$ (6, 7). Historically, this has been assumed to be the case for the phases of Pu (41) (Fig. 4*D*); the small assumed values of $\gamma_{\mathrm{el}}$ are typically those predicted by density functional theory calculations.

The larger-valued temperature-dependent $C_{\mathrm{el}}\left(T\right)/T$ curves for *α*-Pu and *δ*-Pu_0.98_Ga_0.02_ that we obtain in Fig. 2 *E* and *F* do not change the fact that $S_{\mathrm{el}}\left(T\right)\ll S_{\mathrm{ph}}\left(T\right)$. However, because the electronic entropy of *α*-Pu is much lower than that of *δ*-Pu_0.98_Ga_0.02_, the difference in electronic entropy $\Delta S_{\mathrm{el}}$ between the *α* and *δ* phases becomes comparable to the difference in phonon entropy $\Delta S_{\mathrm{ph}}$ (Fig. 4*B*); this is very different from the phonon-dominated entropy difference that had previously been assumed (Fig. 4*E*). The detailed form of $C_{\mathrm{el}}\left(T\right)/T$ is therefore of crucial importance. Fig. 4*B* shows that $\Delta S_{\mathrm{el}}>\Delta S_{\mathrm{ph}}$ once T≳ 190 K. Hence, rather than being dominated by phonons as has been assumed in Fig. 4*F*, in Fig. 4*C*, we find the difference in electronic entropy to become increasingly dominant with increasing temperature. Since the *α* to *δ* structural transformations take place above room temperature, they must occur in a regime where the difference in electronic entropy dominates.

There are two leading contributions to the steadily increasing $\Delta S_{\mathrm{el}}-\Delta S_{\mathrm{ph}}$ beyond T≳ 190 K in Fig. 4*C*. One of these is the large thermal expansivity of *α*-Pu relative to *δ*-Pu_0.98_Ga_0.02_. The other is the broader width of the peak in $C_{\mathrm{el}}/T$ in *δ*-Pu_0.98_Ga_0.02_ relative to *α*-Pu. However, it should be noted that while the difference in electronic entropy (shown in Fig. 4*B*) becomes dominant with rising temperature above room temperature, it is expected to eventually reach saturation, and be accompanied by smaller electronic contributions to $C_{\mathrm{el}}/T$. This prediction is conditional upon the continued adherence of the $C_{\mathrm{el}}/T$ curves to the functional form of a Schotte–Schotte anomaly above 300 K. The tendency of $S_{\mathrm{el}}$ to saturate with increasing temperature in Pu (see, for e.g., Fig. 1*C*) is typical of narrow band strongly correlated electron systems, and is a factor that needs more careful consideration in the development of accurate models of the equation of state.

## Materials and Methods

### Sample Preparation.

The double electro-refined *α*-Pu sample had a nominal isotopic composition of 0.02% 238 Pu, 93.6% 239 Pu, 5.9% 240 Pu, 0.44% 241 Pu, and 0.04% 242 Pu (58). Prior to our calorimetry measurements, the material had aged 6 y, during which time it would have accumulated radiolytically generated impurities, such as H, U, Am, and He. Other impurities, such as Al and Fe, were typically at the 100 parts per million level.

### Specific Heat Measurements.

Specific heat measurements were made on an *α*-Pu sample of smaller mass 5.6 mg than prior measurements (31) so as to reduce the effects of self-heating. Similar-sized samples had previously been used for specific heat measurements on *δ*-Pu_0.98_Ga_0.02_ (25, 47). The measurements were made using a standard quantum design physical properties measurement system (PPMS), with the same addendum (and a similar amount of grease) having been used for the *δ*-Pu_0.98_Ga_0.02_ measurements. While the addendum, inclusive of Apiezon N grease, is measured each time prior to sample mounting, a small difference in the amount of grease for each sample introduces an additional experimental uncertainty. It should be noted, however, that the heat capacity of Apiezon N grease consists of a sharp peak at T≈ 300 K (59), which is quite different from the form of the specific heat of Pu.

For the *δ*-Pu_0.98_Ga_0.02_ sample, it had been noted that the sample length underwent a small reduction at ≈150 K of δl/l≈ 0.15% (47), which, given the smaller atomic volume of $\alpha^{\prime }$-Pu phase, is estimated to correspond to 2.3% of the sample (by volume) transforming into $\alpha^{\prime }$.

### Robustness of the Peak in *C*_el_/*T*.

Given that $C_{\mathrm{ph}}/T$ is approximately four times larger than $C_{\mathrm{el}}/T$ at their respective peak values, any overestimation of $C_{p}/T$ during measurements or underestimation of $C_{\mathrm{ph}}/T$ will ultimately influence the line-shape of $C_{\mathrm{el}}/T$. However, we find several factors that lead us to conclude that the peaks in $C_{\mathrm{el}}/T$ are robust against overestimates of $C_{p}/T$ and underestimates of $C_{\mathrm{ph}}/T$. It is crucial to note that $C_{\mathrm{ph}}$ depends only slowly on *T* at T≳ 50 K and is therefore no longer strongly dependent on the choice of phonon DOS. After performing the phonon subtraction in *α*-Pu, $C_{\mathrm{el}}/T$ approaches zero at T≳ 200 K, implying little room for an overestimated $C_{p}/T$ or an underestimated $C_{\mathrm{ph}}/T$. In the case of *δ*-Pu_0.98_Ga_0.02_, $C_{\mathrm{el}}/T$ continues to have a finite value at T≳ 200 K, which leaves more room for a subtraction error.

To verify the reproducibility of $C_{\mathrm{el}}/T$ in *δ*-Pu, two approaches were employed. First, an entirely different phonon density of states measurement was used to compute $C_{\mathrm{ph}}/T$ (shown by purple curves) in place of the one from ref. 26 (black curves), as depicted in Fig. 2 *D* and *F* and discussed in the main text. Second, the $C_{\mathrm{ph}}/T$ subtraction was repeated using entirely different measurements of $C_{p}/T$, as shown in figures 2 A–C of ref. 25 and SI Appendix, figure 7B of ref. 25. This included measurements of *δ*-Pu stabilized with larger Ga amounts and *δ*-Pu stabilized with Am. In all cases, a clear peak in $C_{\mathrm{el}}/T$ was evident.

The above findings indicate that the peaks in $C_{\mathrm{el}}/T$ are robust against typical experimental uncertainties in both $C_{p}$ and the phonon density of states. It is important to note, however, that the treatment of phonon softening was less rigorous in ref. 25 than in the present manuscript.

The most significant error in subtracting $C_{\mathrm{ph}}/T$ is primarily systematic and arises from the neglect of phonon softening. This omission results in an overestimated $C_{\mathrm{ph}}/T$ at temperatures below ∼100 K, leading to forms of $C_{\mathrm{el}}/T$ (depicted by the green and gray curves in Fig. 2 *E* and *F*) that include negative values. These curves cannot be accounted for by a physical model of the electronic DOS (i.e., one where $D_{\mathrm{el}}\left(\varepsilon \right)$ in Eq. **3** is positive for all *ε*). Nevertheless, it is noteworthy that even when we do not consider phonon softening, peaks in $C_{\mathrm{el}}/T$, although strongly distorted, continue to be obtained. Only when we account for phonon softening do we derive forms for $C_{\mathrm{el}}/T$ that can be represented by a physical model. This is equally true for *α*-Pu and *δ*-Pu_0.98_Ga_0.02_, $C_{\mathrm{el}}/T$.

### The Schotte–Schotte Anomaly.

A single energy level separated from the chemical potential *μ* by a gap Δ gives rise to a Schottky anomaly (60) in the specific heat. When this level is broadened by a finite lifetime to give rise to a Lorentzian-shaped feature in the electronic DOS, the result is a feature with the same functional form as a Schotte–Schotte anomaly in the specific heat (30). The Schotte–Schotte model was originally introduced to account for the specific heat of certain *f*-electron compounds in strong magnetic fields, where it is postulated that a simple Kondo resonance in the shape of a Lorentzian undergoes Zeeman splitting. The specific heat of the anomaly is given by

[3] $$\begin{array}{c} C_{\mathrm{el}}\left(T\right)\approx \frac{R}{k_{B}}\frac{\partial }{\partial T}[\int_{-\infty }^{\infty }\varepsilon D_{\mathrm{el}}\left(\varepsilon \right)f\left(\varepsilon /T\right)d\varepsilon ], \end{array}$$

where the electronic DOS

$$\begin{array}{c} D_{\mathrm{el}}\left(\varepsilon \right)=\frac{\eta \Gamma }{\pi ({\left(\varepsilon -\mu \pm \Delta \right)}^{2}+\Gamma^{2})} \end{array}$$

has the form of a Lorentzian of width Γ, *ε* is the energy and f(ε/T) is the Fermi–Dirac distribution function. Specific heat curves with the same functional form as a Schotte–Schotte anomaly are common in valence fluctuating systems because they often have prominent features in the electronic DOS that are lifetime-broadened and often separated from *μ* by a small energy gap (15, 54).

Note that *η* is the number of electrons per atomic site contributing to the resonance feature in the DOS, or equivalently its overall spectral weight. Since only *f* electrons are expected to yield narrow electronic bands, *η* primarily reflects the total *f*-electron spectral weight close to the Fermi surface.

## Data Availability

All study data are included in the main text.
